# Atraumatic Retrieval of a Fractured Implant Screw in a Maxillary Locator-Retained Overdenture: A Longitudinal Case Report

**DOI:** 10.7759/cureus.110170

**Published:** 2026-06-03

**Authors:** Abdulaziz Y Alwaqyan

**Affiliations:** 1 Advanced Education in General Dentistry, Kuwait Oil Company Hospital, Al Ahmadi, KWT

**Keywords:** adhesive-assisted retrieval, cement-retained crown, dental implant complications, fractured screw removal, implant abutment screw fracture, implant-supported removable overdenture, minimally invasive technique, screw retrieval technique, ultrasonic instrumentation

## Abstract

Fracture of implant prosthetic screws is a significant mechanical complication that may compromise implant stability, prosthetic function, and long-term restorative success. Retrieval of fractured screw fragments can be particularly challenging in implant overdenture patients because of limited access, prosthetic wear, and the risk of damaging the implant’s internal connection during retrieval procedures. This report describes the conservative management of a fractured implant screw associated with a maxillary locator-retained overdenture in a medically compromised elderly patient with a prolonged history of prosthetic maintenance complications.

A 67-year-old female patient with a history of diabetes mellitus, heavy smoking, poor oral hygiene, and long-term implant overdenture therapy underwent staged maxillary and mandibular implant rehabilitation. Over several years of follow-up, repeated prosthetic complications were documented, including overdenture instability, worn locator attachments, recurrent retention loss, relining procedures, soft tissue overgrowth, and ongoing prosthetic adjustments. The patient subsequently presented with a fracture of a prosthetic screw associated with the maxillary overdenture attachment system.

Following prosthetic disassembly and conservative soft tissue exposure using a laser-assisted approach, the fractured screw fragment was mobilized using ultrasonic instrumentation in a counterclockwise direction. A flexible adhesive-assisted retrieval technique was then utilized to atraumatically engage and remove the fractured fragment while minimizing the risk of damage to the implant’s internal threads and connection. The fractured fragment was successfully retrieved without complications, and the implant remained intact and functional without the need for implant removal or aggressive surgical intervention.

This case highlights the biomechanical and maintenance-related challenges associated with long-term implant overdenture therapy in medically compromised patients and demonstrates the clinical usefulness of minimally invasive screw retrieval techniques for preservation of implant function and prosthetic continuity.

## Introduction

Fracture of implant prosthetic screws is considered a relatively uncommon but clinically significant mechanical complication that may compromise prosthetic stability, implant function, and long-term treatment success [1,2]. Management becomes particularly challenging when the fractured fragment remains lodged within the implant body, especially in implant-retained overdenture restorations exposed to prolonged functional loading, repeated insertion and removal cycles, prosthetic wear, and recurrent maintenance procedures over extended periods of service.

Retrieval of fractured prosthetic screw fragments is often technique-sensitive and may require specialized instrumentation or invasive interventions. Various management approaches have been described in the literature, including manufacturer-specific retrieval kits, ultrasonic instrumentation, modified rotary instruments, adhesive-assisted techniques, and surgical implant removal in severe cases [3-7]. However, these approaches may be associated with complications such as damage to the implant’s internal connection, compromised restorability, limited tactile control, overheating, or inadvertent apical displacement of the fractured component [4,5].

The complexity of management further increases in situations involving soft tissue overgrowth, limited restorative access, medically compromised patients, or implants supporting removable overdenture prostheses with long-term functional use. In these situations, preservation of the implant’s internal threads and avoidance of additional iatrogenic damage are essential for maintaining implant function and prosthetic continuity [1,8-10].

Minimally invasive retrieval strategies aimed at preserving implant integrity have therefore gained increasing clinical importance. Conservative soft tissue management using laser-assisted techniques may improve visualization and access while minimizing bleeding and tissue trauma. In addition, sequential atraumatic approaches combining manual manipulation, ultrasonic micro-vibration, and adhesive-assisted retrieval techniques may facilitate the removal of fractured prosthetic components while reducing the risk of internal implant damage.

This report describes the conservative retrieval of a fractured prosthetic screw associated with a maxillary implant-retained overdenture supported by a Straumann Bone Level Tapered (BLT) implant (Institut Straumann AG, Basel, Switzerland) using a minimally invasive multimodal retrieval approach. The technique involved Waterlase-assisted soft tissue management, mechanical manipulation with a periodontal probe, controlled ultrasonic instrumentation, and adhesive-assisted retrieval using a Sticky-Ticky tip while preserving implant integrity and prosthetic restorability.

## Case presentation

A 67-year-old female patient presented with a chief complaint of recurrent loosening and reduced retention of a maxillary implant-retained overdenture. Her medical history was significant for diabetes mellitus, heavy smoking, and intermittent low-dose aspirin use. The patient also reported intolerance to Augmentin, while amoxicillin was tolerated without a documented allergy.

The patient had a prolonged history of advanced dental deterioration, multiple retained roots, failing fixed prostheses, poor oral hygiene, and staged dental extractions followed by implant-assisted prosthetic rehabilitation. The maxillary implant-retained overdenture had been in function for approximately five years before the patient presented with recurrent loosening and reduced retention. Clinical records documented repeated prosthetic maintenance visits during this period, including denture relining, overdenture attachment adjustment, locator insert replacement, recurrent loss of retention, soft tissue overgrowth, and laser-assisted soft tissue management around the locator attachment components. At the time of presentation, the occlusion of the overdenture prosthesis was clinically assessed. No gross occlusal discrepancy or obvious premature contact was detected; however, prosthetic wear consistent with long-term functional use was noted. Based on the available clinical records and patient history, there was no documented previous replacement of the fractured prosthetic screw since the fabrication of the prosthesis.

Radiographic evaluation demonstrated a maxillary locator-retained implant overdenture supported by multiple implants with maintained implant positioning and surrounding peri-implant bone support (Figure 1).

**Figure 1:**
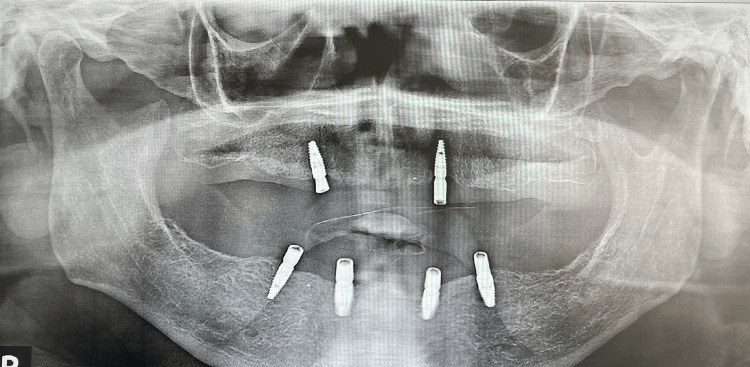
Panoramic radiographic evaluation demonstrating maxillary and mandibular implant-supported prosthetic rehabilitation Preoperative panoramic radiograph demonstrating the existing implant-supported overdenture rehabilitation with maintained implant positioning and surrounding peri-implant bone support.

Clinical and radiographic examination identified a fractured prosthetic screw associated with the maxillary right second molar implant region. The fractured screw fragment remained lodged within the implant connection complex, while the implant itself remained clinically stable, osseointegrated, and radiographically restorable (Figure 2).

**Figure 2:**
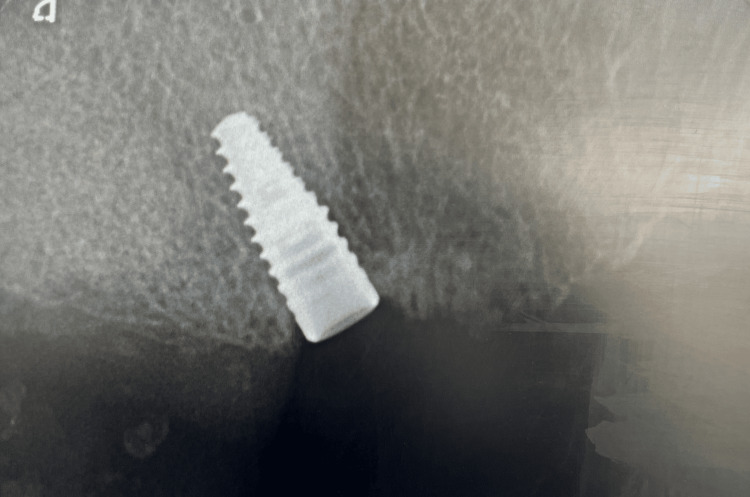
Periapical radiographic view of the fractured prosthetic screw within the maxillary implant connection Preoperative periapical radiograph demonstrating the fractured prosthetic screw fragment lodged within the internal connection of the maxillary right posterior Straumann BLT implant supporting the overdenture attachment system. The implant fixture remained osseointegrated with maintained surrounding peri-implant bone support. BLT: Bone Level Tapered

The affected implant was identified as a Straumann Bone Level Tapered (BLT) implant (Institut Straumann AG, Basel, Switzerland) measuring 4.1 mm × 10 mm with a CrossFit internal conical connection design supporting a locator overdenture attachment system. The prosthetic screw had originally been tightened according to the manufacturer’s recommended torque specifications. Based on the patient’s clinical history, repeated insertion and removal cycles associated with overdenture function, oral hygiene maintenance, long-term prosthetic service, and posterior maxillary functional loading were considered contributing factors to prosthetic screw fatigue and eventual fracture.

Given the patient’s complex prosthetic history and the importance of preserving the existing osseointegrated implant rehabilitation, a conservative minimally invasive retrieval approach was selected to avoid damage to the implant connection and prevent unnecessary implant removal.

Initial clinical assessment confirmed the presence of the fractured prosthetic screw within the implant connection. Soft tissue overgrowth surrounding the locator attachment was conservatively managed using a Waterlase laser system (Biolase, Irvine, CA) to improve visualization and access while minimizing tissue trauma and bleeding (Figure 3 and Figure 4).

**Figure 3:**
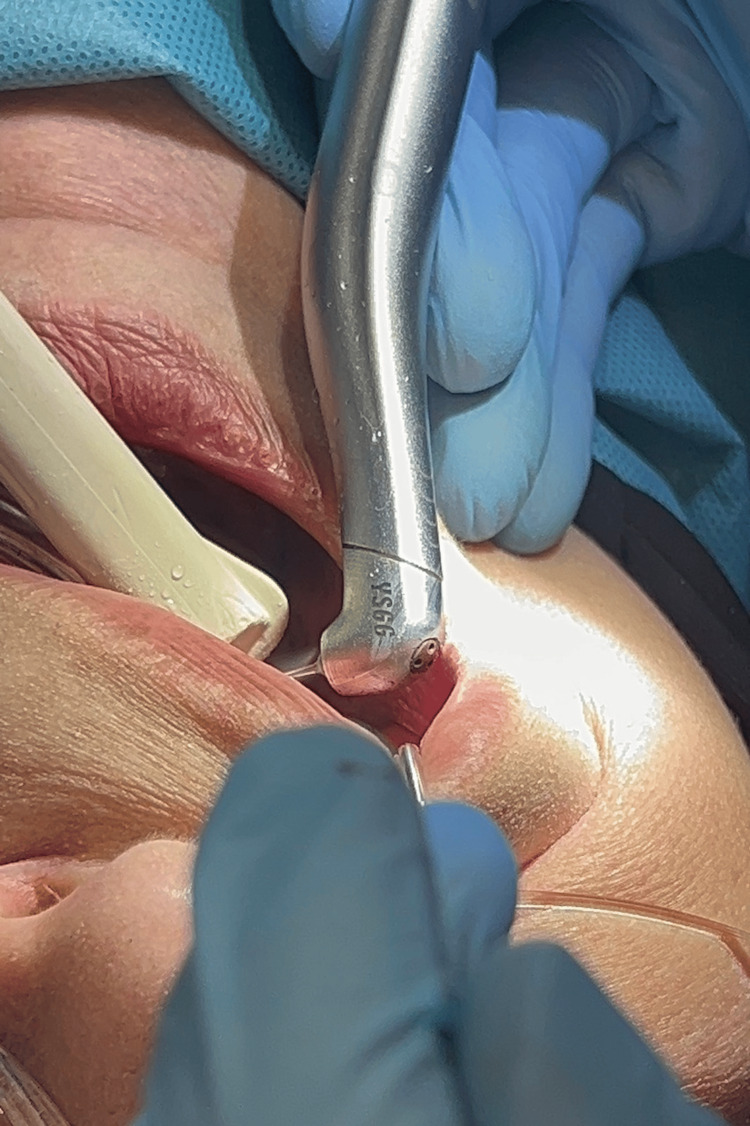
Laser-assisted soft tissue management and initial clinical access to the fractured prosthetic screw site Intraoperative clinical photograph demonstrating conservative soft tissue management around the maxillary overdenture attachment site using a Waterlase laser system to improve visualization and access to the fractured prosthetic screw fragment while minimizing tissue trauma and bleeding prior to retrieval procedures.

**Figure 4:**
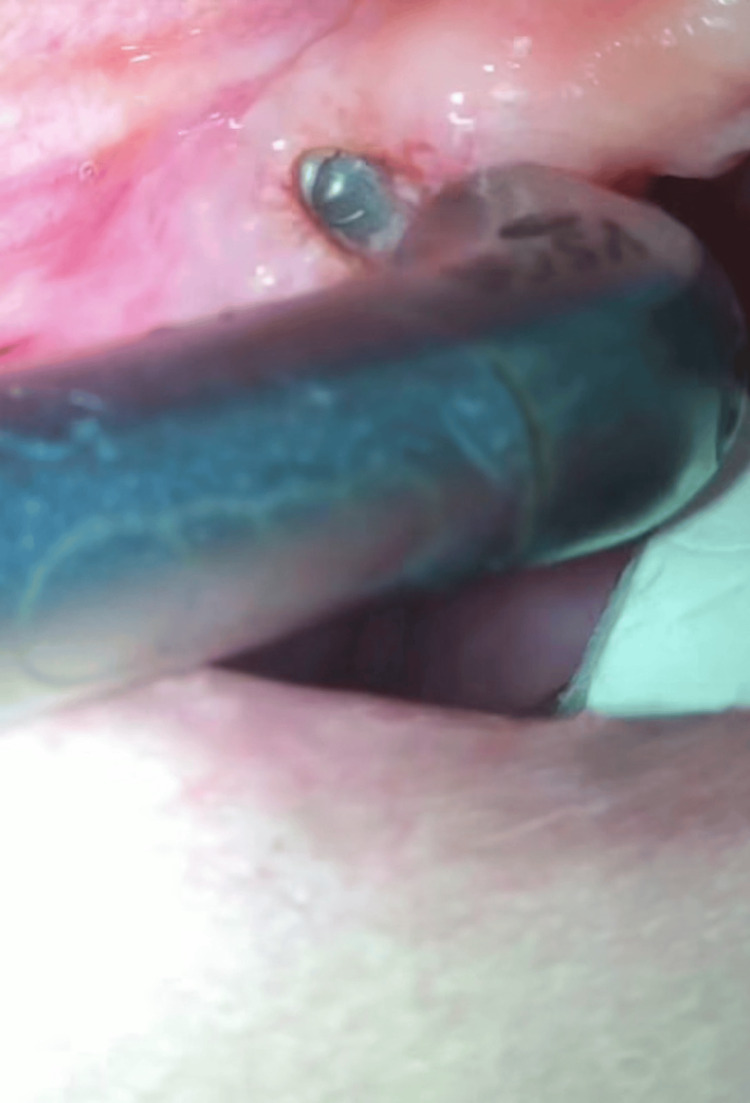
Laser-assisted soft tissue management and initial clinical access to the fractured prosthetic screw site

Ultrasonic instrumentation was subsequently applied in a controlled counterclockwise direction under magnification to conservatively trough around the fractured screw fragment and generate micro-vibratory movement to facilitate mobilization while preserving the integrity of the implant connection (Figure 5).

**Figure 5:**
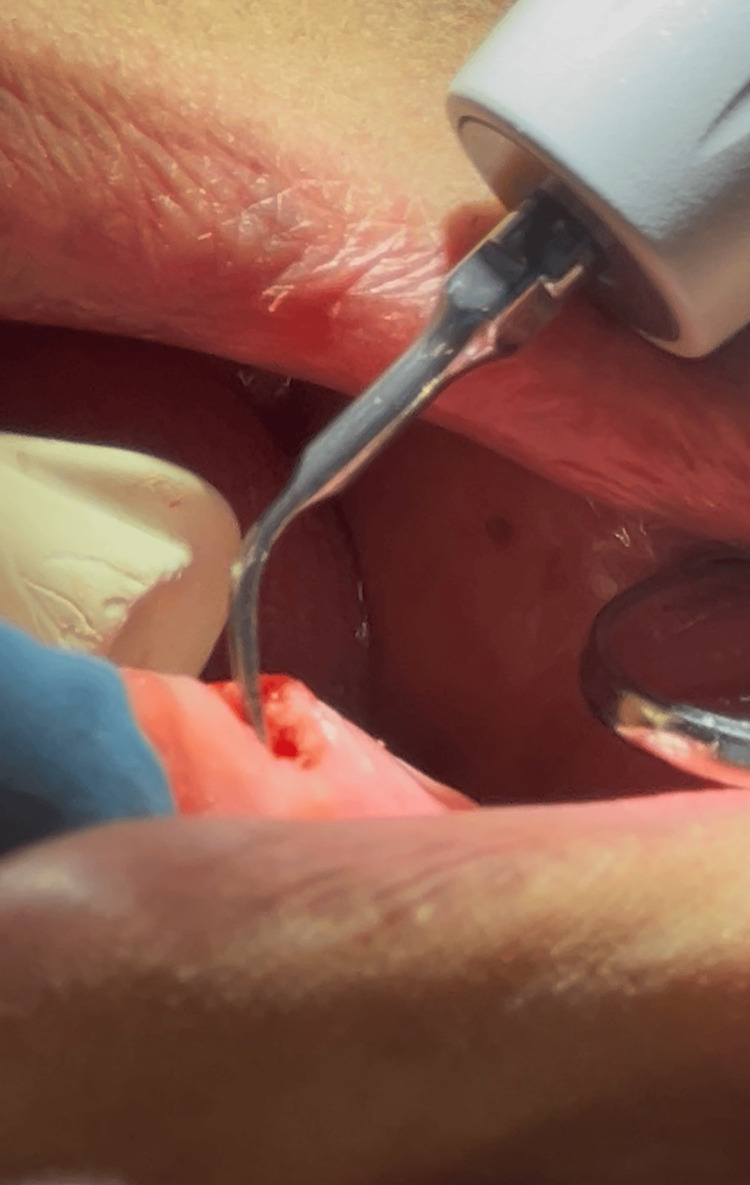
Ultrasonic mobilization of the fractured prosthetic screw fragment Intraoperative clinical photograph demonstrating controlled ultrasonic instrumentation applied in a counterclockwise direction to conservatively trough around and mobilize the fractured prosthetic screw fragment within the implant connection while preserving the integrity of the internal implant threads.

Following partial mobilization and coronal exposure of the fractured fragment, cyanoacrylate adhesive was carefully applied using a flexible micro-applicator “Sticky-Ticky” tip to atraumatically engage the loosened screw fragment (Figure 6).

**Figure 6:**
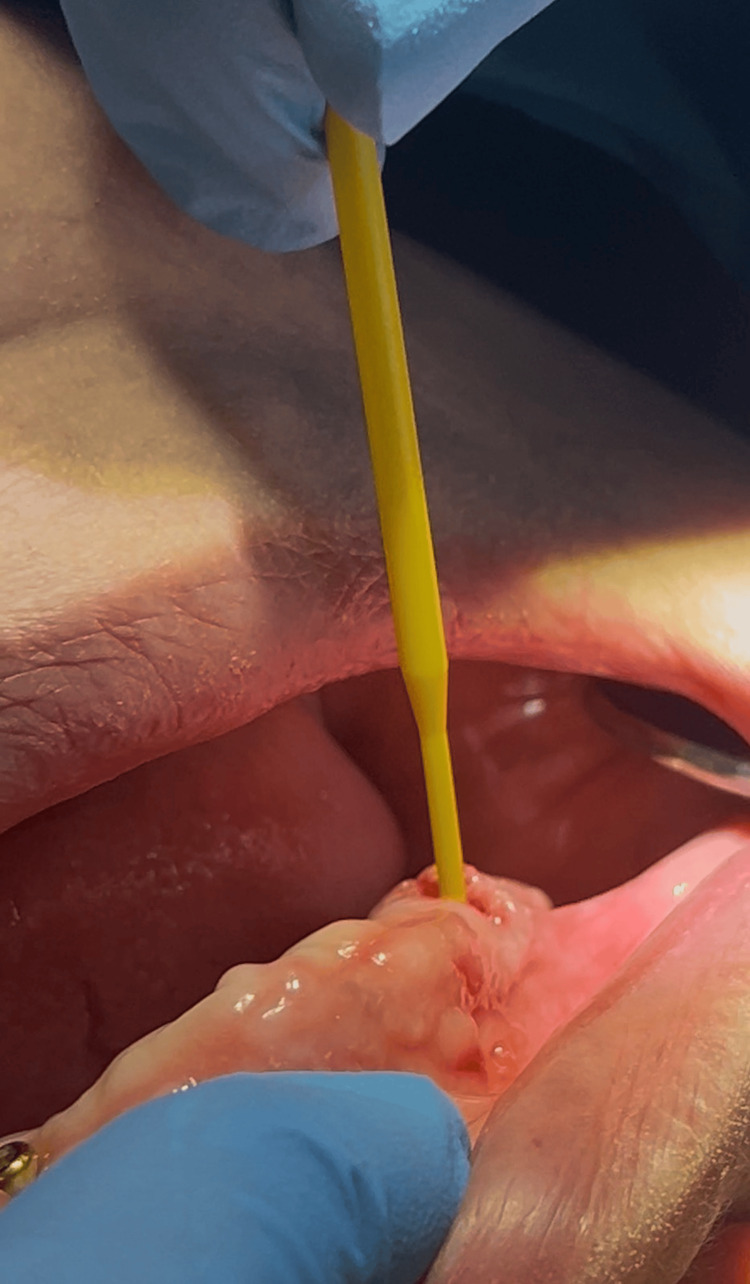
Adhesive-assisted engagement of the fractured prosthetic screw fragment using a flexible micro-applicator tip Intraoperative clinical photograph demonstrating application of cyanoacrylate adhesive using a flexible micro-applicator “Sticky-Ticky” tip to atraumatically engage the partially mobilized fractured prosthetic screw fragment for controlled retrieval from the implant connection.

The adhesive-assisted applicator successfully engaged the fractured prosthetic screw fragment, allowing controlled retrieval from the implant connection without additional implant damage (Figure 7 and Figure 8).

**Figure 7:**
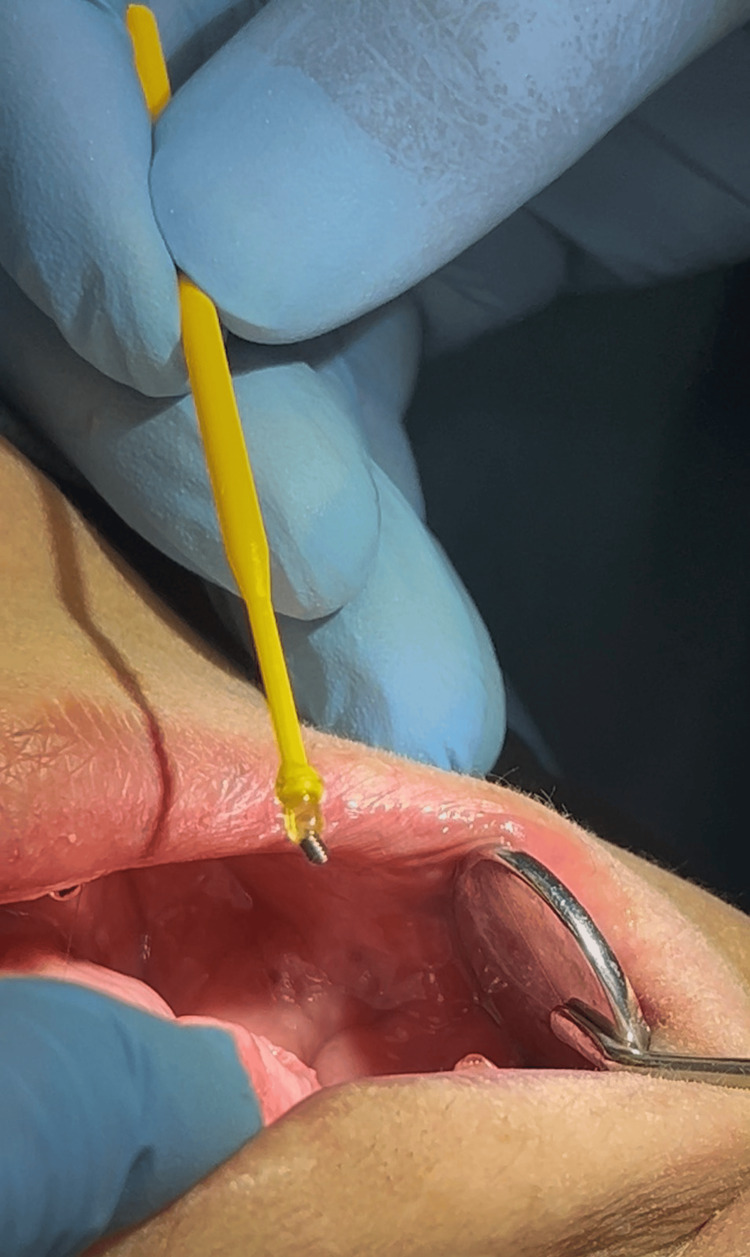
Atraumatic retrieval of the fractured prosthetic screw fragment from the implant connection Intraoperative clinical photograph demonstrating successful adhesive-assisted retrieval of the fractured prosthetic screw fragment using the flexible “Sticky-Ticky” micro-applicator tip following ultrasonic mobilization while preserving the integrity of the implant’s internal connection.

**Figure 8:**
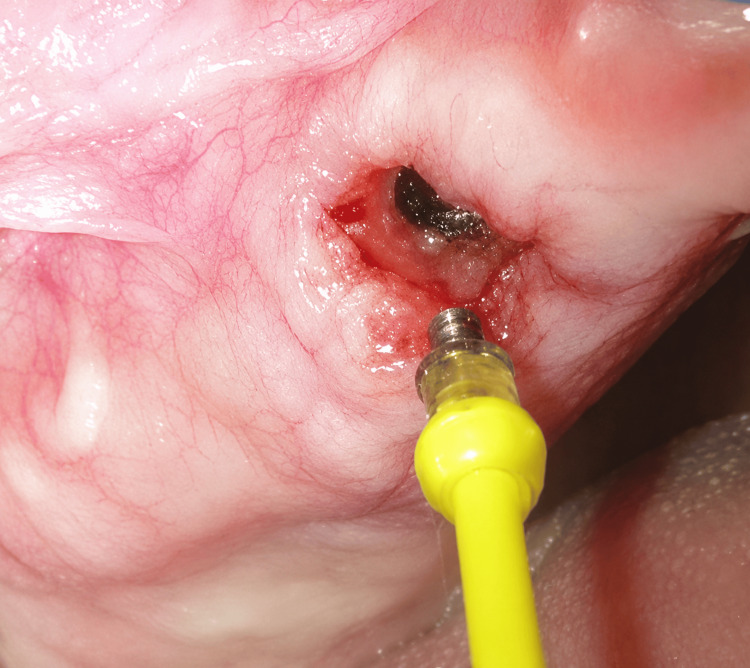
Atraumatic retrieval of the fractured prosthetic screw fragment from the implant connection

Clinical evaluation following retrieval demonstrated preservation of the implant connection integrity without visible deformation or significant internal damage (Figure 9).

**Figure 9:**
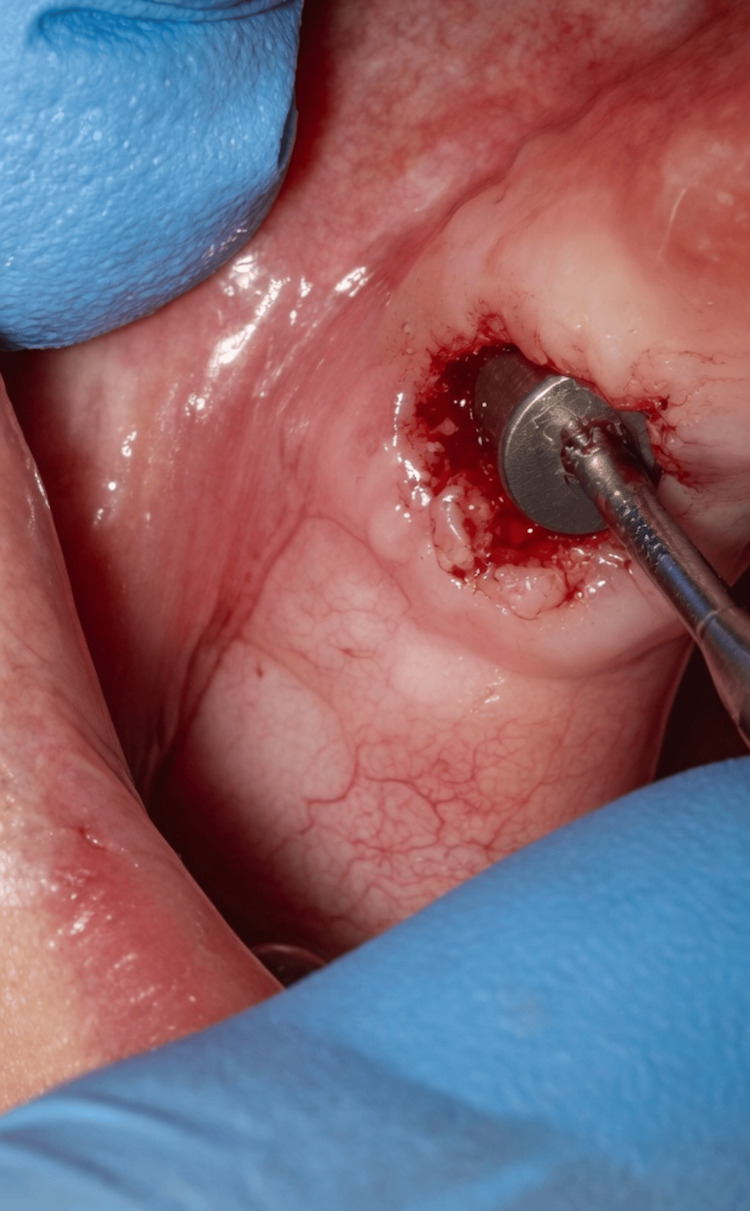
Clinical evaluation of the implant connection following fractured screw retrieval Intraoperative clinical photograph demonstrating preservation of the internal implant connection and surrounding peri-implant soft tissues following atraumatic retrieval of the fractured prosthetic screw fragment, without visible deformation or significant internal damage to the implant interface.

Postoperative radiographic evaluation confirmed maintenance of the implant fixture and surrounding peri-implant structures without evidence of implant fracture, loss of osseointegration, or significant peri-implant complications (Figure 10).

**Figure 10:**
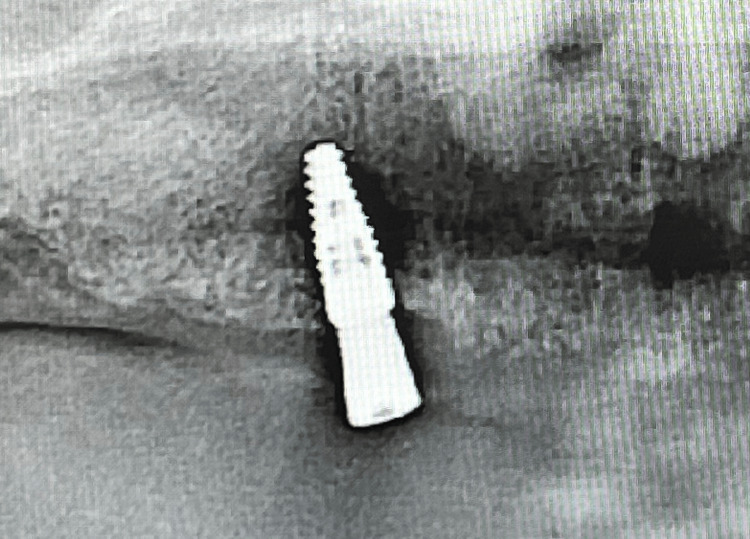
Postoperative radiographic evaluation of the implant fixture Postoperative periapical radiograph demonstrating the implant fixture in the maxillary right posterior region with maintained fixture integrity and surrounding peri-implant bone support. No radiographic evidence of implant fracture, loss of osseointegration, or significant peri-implant pathology is observed.

A replacement prosthetic screw and overdenture attachment component were subsequently inserted and secured according to the manufacturer’s recommended torque specifications. The patient was instructed regarding overdenture maintenance, oral hygiene measures, smoking reduction, routine prosthetic follow-up care, and periodic maintenance visits to minimize future prosthetic complications.

## Discussion

Fracture of implant prosthetic screws represents a relatively uncommon but clinically significant mechanical complication that may compromise the long-term stability, retention, and function of implant-supported prostheses [1,2,9]. Management becomes particularly challenging when the fractured fragment remains lodged within the implant body, especially in implant-retained overdenture attachment systems, where access and visualization may be limited. Preservation of the osseointegrated implant and maintenance of the implant’s internal prosthetic connection remain the primary treatment objectives, as implant removal may increase surgical morbidity, treatment complexity, patient discomfort, and financial burden.

Several contributing factors associated with prosthetic screw fracture have been reported in the literature, including cyclic occlusal loading, repeated insertion and removal of removable overdentures, prosthetic wear, posterior functional loading, screw fatigue, parafunctional activity, and inadequate passive fit [1,2,6]. In medically compromised patients with long-term prosthetic maintenance histories, these biomechanical and functional stresses may further increase the risk of mechanical complications.

Multiple retrieval techniques for fractured implant screws have been described, including manufacturer-specific retrieval kits, explorers, modified rotary instruments, endodontic files, hemostats, ultrasonic instrumentation, adhesive-assisted techniques, and surgical implant removal in severe cases [3-7]. Ultrasonic instrumentation is commonly utilized because of its ability to generate vibration and facilitate controlled counterclockwise mobilization of fractured screw fragments. Nevertheless, despite its clinical effectiveness, ultrasonic instrumentation may still present limitations, including inadequate control during retrieval, overheating, apical displacement of the fragment, and inadvertent damage to the implant’s internal threads or connection geometry [3,5].

In the present case, a conservative multimodal retrieval approach was successfully employed to preserve the existing osseointegrated implant restoration. The affected implant was a Straumann Bone Level Tapered (BLT) implant measuring 4.1 mm × 10 mm with a CrossFit internal conical connection supporting a maxillary locator-retained overdenture attachment system. Initial management included Waterlase-assisted soft tissue removal to conservatively expose the implant connection and improve visualization while minimizing tissue trauma and bleeding. Careful manual manipulation using periodontal instrumentation was subsequently performed to assess fragment mobility prior to controlled ultrasonic activation.

Ultrasonic instrumentation was then utilized in a counterclockwise direction under magnification to conservatively trough around the fractured prosthetic screw fragment and facilitate gradual mobilization while preserving implant connection integrity. Following partial coronal exposure and mobilization of the fractured component, a flexible adhesive-assisted micro-applicator (“Sticky-Ticky” tip) with cyanoacrylate adhesive was used to atraumatically engage and retrieve the loosened fragment from the implant body.

This combined minimally invasive approach allowed successful retrieval of the fractured prosthetic screw while preserving the implant’s internal threads, maintaining implant stability, and avoiding unnecessary implant removal or additional surgical intervention. The present report highlights the clinical importance of conservative retrieval strategies in managing prosthetic complications associated with implant-retained overdenture systems.

## Conclusions

Fracture of implant prosthetic screws is a clinically significant mechanical complication that may compromise the stability and function of implant-supported overdentures. Preservation of the existing osseointegrated implant and its internal connection remains the primary treatment objective during management of these complications.

In the present case, a conservative minimally invasive multimodal retrieval approach combining Waterlase-assisted soft tissue management, periodontal instrumentation, controlled ultrasonic counterclockwise mobilization, and adhesive-assisted retrieval successfully enabled atraumatic removal of a fractured prosthetic screw fragment from a Straumann Bone Level Tapered (BLT) 4.1 mm × 10 mm implant supporting a maxillary locator-retained overdenture. The described technique preserved implant connection integrity while avoiding unnecessary implant removal or additional surgical intervention. This conservative approach may provide clinicians with a practical and clinically effective option for the management of similar implant prosthetic complications, particularly in medically compromised or long-term overdenture patients.
